# Genomic and epigenomic landscape of ADPKD: towards precision diagnostics and tailored treatments

**DOI:** 10.3389/freae.2026.1699528

**Published:** 2026-02-06

**Authors:** Amankeldi A. Salybekov, Aiman Kinzhebay, Sumi Hidaka, Shuzo Kobayashi

**Affiliations:** 1 Kidney Diseases and Transplant Center, Shonan Kamakura General Hospital, Kamakura, Japan; 2 Cell and Gene Therapy Department, Regenerative Medicine Division, Qazaq Institute of Innovative Medicine, Astana, Kazakhstan

**Keywords:** AI, autosomal dominant polycystic kidney diseases, cystogenesis, diagnostics, epigenetics

## Abstract

Autosomal dominant polycystic kidney disease (ADPKD) represents one of the most prevalent hereditary renal disorders, affecting an estimated 12.5 million individuals globally and characterized by progressive cyst formation in both kidneys. While mutations in *PKD1* and *PKD2* genes account for most cases, recent research has identified rare causative genes as contributing factors in genetically unresolved cases. The review examines the molecular mechanisms of cystogenesis, highlighting how genetic predisposition interacts with epigenetic modifications, including DNA methylation patterns, histone alterations, and non-coding RNAs (miRNAs and piRNAs). Advanced diagnostic approaches, from conventional imaging to AI-assisted cyst segmentation and long-read sequencing technologies, are evaluated for their clinical utility. Long-read sequencing platforms increase diagnostic yield by up to 25% in previously unsolved cases, while AI-enhanced imaging provides superior accuracy in disease progression monitoring. Finally, the paper explores emerging precision medicine strategies, including targeted therapies directed at specific molecular pathways, risk stratification algorithms, and personalized treatment approaches based on individual genetic and epigenetic profiles. This integration of genomic and epigenomic insights provides a foundation for improved prognostic models, early biomarkers, and tailored therapeutic interventions for ADPKD patients.

## 1 Introduction

Autosomal dominant polycystic kidney disease (ADPKD) is a heritable genetic condition resulting in bilateral renal cysts. As a progressive disorder, it is also associated with kidney enlargement, hypertension, acute and chronic pain, hematuria, cyst infection, nephrolithiasis, and renal failure (Cornec-Le Gall et al., 2019). It often leads to chronic kidney disease (CKD) and consequently to end-stage kidney disease (ESKD) (Reiterová and Tesař, 2022). However, the scope of this systemic disease extends past the bounds of kidneys, affecting multiple organs, including the liver cysts, cardiac valvular disease like VHD, and intracranial aneurysms, with prevalence of 83%, 25%, and 10% respectively (Suzuki et al., 2023). Affecting approximately 12.5 million people worldwide, ADPKD is one of the leading causes of ESKD, accounting for up to 10% of all cases (Chapman et al., 2015). Disease progression varies, but up to 70% of individuals with ADPKD reach ESKD by the age of 70, necessitating renal replacement therapy such as dialysis or kidney transplantation (Neumann et al., 2013). As a result, ADPKD patients make up approximately 10% of those undergoing dialysis or transplantation programs (Reiterová and Tesař, 2022).

ADPKD is a complex disorder driven by both genetic mutations and epigenetic modifications, necessitating a multi-faceted approach to fully understand its pathogenesis. It is a monogenic disorder that exhibits variable expressivity, meaning that disease severity can differ significantly among affected individuals. The condition is primarily caused by mutations in *PKD1* and *PKD2*, whose protein products play critical roles in renal tubular function and cyst regulation. However, genetic mutations alone do not fully account for the variability in disease severity, suggesting a crucial role for epigenetic regulation in modifying disease progression. DNA methylation, histone modifications (Yan et al., 2025), and non-coding RNAs (Zhou and Li, 2021) have been implicated in influencing gene expression patterns that contribute to cyst proliferation, inflammation, and fibrosis. Moreover, somatic mutations and mosaicism (Devuyst and Pei, 2020; Hopp et al., 2020) further complicate the disease landscape, as different regions of the kidney may harbor varying genetic alterations. Despite these complexities, tolvaptan remains the only Food and Drug Administration (FDA)-approved medication for slowing disease progression (Torres et al., 2012; Torres et al., 2017), underscoring the need for further research into more effective and targeted therapeutic strategies, especially in sight of the reported long term side effects.

The integration of genomic and epigenomic insights is therefore essential for developing more accurate prognostic models, identifying biomarkers for early diagnosis, and tailoring precision therapies that target both genetic and epigenetic pathways. Future research must leverage multi-omics approaches, including transcriptomics and metabolomics, to fully delineate the interplay between genetic predisposition and environmental influences in ADPKD pathophysiology. This review aims to summarise the current understanding of genomic mutations and epigenomic modifications that contribute to ADPKD onset and progression, as well as highlighting the practical use of the findings in diagnostics and therapy.

## 2 The role of somatic and germline mutations in ADPKD pathogenesis

Mutations in PKD1 and PKD2 are the most common ADPKD causes accounting for 78% and 15% of disease pedigrees respectively (Table 1) (Cornec-Le Gall et al., 2018). They encode Polycystin-1 (PC1) and polycystin-2 (PC2), respectively, which transmembrane proteins that are widely present in epithelial cells and are also found in endothelial cells, smooth muscle, and cardiomyocytes (Cornec-Le Gall et al., 2019). However, the area of interest is the primary cilia of tubular epithelial cells, where they play a critical role in mechanosensation and calcium signalling. Mutations in these genes disrupt normal cellular signalling, leading to abnormal proliferation, fluid secretion, and cyst formation in the kidneys. To date, over 1,500 distinct variants of *PKD1* and over 250 variants of *PKD2* have been documented in the ADPKD mutation database (Cornec-Le Gall et al., 2018; ADPKD Variant Database, 2026). Truncating variants (including nonsense, frameshift, canonical splice-site, and large deletions) are overwhelmingly deemed pathogenic or likely pathogenic and strongly associated with early-onset, rapidly progressive disease (Yang et al., 2023). Non-truncating and missense variants, by contrast, frequently fell into the variant of uncertain significance (VUS) category, reflecting both the structural complexity of *PKD1* (with its duplicated pseudogenes) and the incomplete functional annotation of its domains (Yang et al., 2023). Importantly, even among missense variants, several recurrent substitutions located in conserved PKD repeats or transmembrane domains have now accumulated sufficient evidence for pathogenic classification (Yang et al., 2023). Although, the function of these two is related, their presence and expression are independent and sometimes only one of them might be pathogenic (Cornec-Le Gall et al., 2018). While *PKD1* and *PKD2* are responsible for the majority of ADPKD cases, additional genes, such as *GANAB*, *DNAJB11, ALG9, IFT140, ALG5*, and *NEK8* have been implicated in atypical or milder forms of the disease (Lemoine et al., 2022).

**TABLE 1:** Genetic origin of ADPKD and other cystic diseases.

Gene	Chromosome	Prevalence, %	Renal phenotype	Extrarenal manifestations	Representative variants (ClinVar)	Variant type	ACMG classification	Mechanism/Function	References
Major ADPKD genes									
*PKD1*	16p13.3	78	Severe ADPKD; early-onset, rapid ESRD (avg. 58 years)	Liver cysts, intracranial aneurysms, cardiac valve disease	c.5014C>T (p.Arg1672*); c.11364del (p.Asn3788Thrfs*42)	Nonsense, frameshift	Pathogenic/Likely pathogenic	Encodes PC1 (500 kDa); regulates Ca^2+^, TSC-mTOR, NFAT, G-protein signaling; high penetrance	Yang et al. (2023); Torres et al. (2025)
*PKD2*	4q22.1	15	Milder, later-onset ESRD (avg. 74.8 years)	Liver cysts, less common vascular lesions	c.1772C>A (p.Ser591*); c.2224C>T (p.Arg742*)	Nonsense	Pathogenic	Encodes PC2 (TRPP2); Ca^2+^-permeable channel in cilia; regulates Wnt/MAPK	Yang et al. (2023); Torres et al. (2025)
Minor ADPKD genes									
*IFT140*	16p13.3	1-2	Milder ADPKD, sometimes liver cysts	Retinal dystrophy (rare), skeletal anomalies	c.634G>A (p.Gly212Ser)	Missense	Likely pathogenic	Intraflagellar transport; ciliary function	Senum et al. (2022); Torres et al. (2025)
*GANAB*	11q12.3	<0.5	Moderate cystic kidney disease, slow ESRD	Liver cysts predominant	c.2810G>A (p.Arg937His); c.3089C>T (p.Pro1030Leu)	Missense	Likely pathogenic	Glucosidase IIα; affects PC1 maturation/localization	Porath et al. (2016); Cordido al. (2017); Senum et al. (2022); Torres et al. (2025)
*DNAJB11*	3q27.3	<0.5	Small kidneys, interstitial fibrosis, late ESRD	Mild hepatic cysts	c.1003C>T (p.Arg335*); c.479del (p.Asn160Metfs*6)	Nonsense, frameshift	Pathogenic	BiP cofactor; affects PC1 trafficking	Cornec-Le Gall et al. (2018); Senum et al. (2022); Torres et al. (2025)
*ALG9*	11p11.2	<0.5	Moderate cystic kidney disease	Hepatic cysts (rare)	c.677G>A (p.Gly226Asp)	Missense	Likely pathogenic	Glycosyltransferase; PC1 glycosylation	Besse et al. (2019); Torres et al. (2025)
*ALG5*	13q14.11	<0.5	Late-onset small cystic kidneys, fibrosis	None known	c.235C>T (p.Arg79*)	Nonsense	Pathogenic	Glycosylation pathway, PC1 processing	Lemoine et al. (2022); Torres et al. (2025)
*NEK8*	17q11.2	<0.5	Variable:early-onset severe or mild ADPKD-like	Congenital heart defects (rare)	c.133C>T (p.Arg45Trp); c.449T>G (p.Ile150Met)	Missense	p.Arg45Trp – Pathogenic; p.Ile150Met – Likely benign	Ciliary kinase; regulates PC2 trafficking	Claus et al. (2023); Torres et al. (2025)

### 2.1 Proposed mechanistic models of cyst initiation

The two-hit hypothesis is a well-established model for explaining cyst development in ADPKD, suggesting that both an inherited germline mutation in *PKD1* or *PKD2* and a subsequent somatic mutation in the remaining functional allele are necessary for cyst formation (Qia et al., 1996; Pei et al., 1999; Brasier and Henske, 1997; Koptides et al., 1998; Koptides et al., 1999). This mechanism aligns with the inactivation of tumor suppressor genes, where loss of heterozygosity (LOH) leads to unregulated cell proliferation and cyst growth (Mallawaarachchi et al., 2016; Badenas et al., 2000). Early investigations into this hypothesis provided evidence of LOH in certain kidney cysts from ADPKD patients, further supporting that cystogenesis results from the inactivation of the normal copy of the gene by a secondary somatic event (Mallawaarachchi et al., 2016; Badenas et al., 2000). Analysis of 211 cysts from seven ADPKD-*PKD1* patients and detected LOH in 13.3% of *PKD1* cysts, reinforcing the idea that ADPKD follows a cellular recessive mechanism (Badenas et al., 2000). Importantly, this LOH was specific to *PKD1*, as no LOH was detected in other chromosomal regions, providing further genetic evidence for the two-hit model (Badenas et al., 2000).

However, somatic mutations were only identified in a subset of tested cysts, raising doubts about whether alternative mechanisms contribute to cyst initiation (Mallawaarachchi et al., 2016). A major obstacle in testing this hypothesis has been the difficulty of sequencing *PKD1*, due to its highly homologous pseudogenes spanning exons 1–33, which complicate accurate detection of mutations (Mallawaarachchi et al., 2016). As a result, it remained unclear whether the lack of detected somatic mutations in some cysts was due to technical limitations or if additional factors were involved in cyst formation (Mallawaarachchi et al., 2016). Recent sequencing advancements, such as the use of unique molecular identifiers (UMIs) for error correction, have significantly improved the sensitivity of detecting low-frequency somatic mutations, reinforcing the role of second-hit mutations in cystogenesis (Mallawaarachchi et al., 2024) The two-hit hypothesis is also strongly supported by the identification of somatic mutations at high allele frequencies (12–80%) in most cysts (90–93%) from ADPKD patients (Tan et al., 2018; Zhang Z. et al., 2021). Using long-range PCR, Sanger sequencing, and exome/genome sequencing, these mutations were primarily found in cells lining large cysts (1.0–15 cm) from end-stage kidneys, suggesting that cysts expand clonally over time (Tan et al., 2018; Zhang Z. et al., 2021). However, since their study focused on larger cysts, it remains uncertain whether somatic mutations occur early in cyst initiation or later during disease progression.

Notably, new findings suggest that while somatic mutations drive cyst formation, haploinsufficiency model, defined as loss of one allele or reduced dosage of functional *PKD1* or *PKD2*, alone may be sufficient to initiate cystogenesis, indicating that not all cysts require a second-hit event (Mallawaarachchi et al., 2024). This concept is supported by experimental models where partial loss of polycystin accelerates cystogenesis, as well as by sequencing studies that fail to detect second-hit mutations in a substantial proportion of cysts (Mallawaarachchi et al., 2024). For instance, a recent deep-sequencing analysis identified somatic PKD1 variants in only ∼58% of cysts, often at low allele frequencies, suggesting that some cysts may arise through dosage insufficiency rather than clonal expansion from a second hit (Mallawaarachchi et al., 2024). While critics argue that technical limitations may explain the ‘missing’ mutations, the haploinsufficiency model underscores the high sensitivity of renal epithelia to polycystin levels and provides a plausible parallel mechanism to the classical two-hit hypothesis (Mallawaarachchi et al., 2024). This raises further questions about whether all cysts strictly follow the two-hit model or if alternative mechanisms contribute to cystogenesis in ADPKD (Torres and Harris, 2019).

Some authors propose the “third hit” hypothesis, suggesting that environmental or non-genetic factors, such as renal injury, ischemic stress, or metabolic disturbances, exacerbate ADPKD pathogenesis (Takakura et al., 2009; Kurbegovic and Trudel, 2016). Chronic renal stressors, including hyperglycemia, oxidative stress, and inflammation, have been shown to worsen disease progression by amplifying cyst expansion through alterations in cell polarity, proliferation, and apoptosis (Takakura et al., 2009; Kurbegovic and Trudel, 2016). These effects are mediated by dysregulated mTOR (mammalian target of rapamycin) activation, JAK-STAT (Janus kinase-signal transducers and activators of transcription) signaling, and hypoxia-inducible factors (HIF-1α) (Takakura et al., 2009). This supports the notion that systemic conditions, such as diabetes or hypertension, may accelerate ADPKD progression even in genetically predisposed individuals. Additionally, kidney injury, such as ischemia-reperfusion injury (IRI), has been identified as a third hit that further accelerates cystogenesis (Kurbegovic and Trudel, 2016). IRI triggers widespread damage, leading to increased PC1 and PC2 expression, dysregulated mTOR and Wnt/β-catenin signaling, and sustained inflammation, all of which contribute to cyst formation (Kurbegovic and Trudel, 2016). These findings suggest that environmental stressors not only worsen ADPKD progression but may even initiate cystogenesis independently of PKD mutations, highlighting the importance of targeting metabolic and injury-related pathways as potential therapeutic strategies.

### 2.2 Signaling pathways in cyst growth and progression

Following the genetic framework of the two-hit hypothesis, the prevailing consensus on mechanism behind PC1 and PC2 cystogenesis is the dose-dependent inhibition, with cysts forming due to their lower concentrations (Cornec-Le Gall et al., 2019). The absence of PC1 or PC2 is believed to result in decreased intracellular calcium levels, which in turn enhances the activity of adenyl cyclase types 5 and 6 while reducing phosphodiesterase 1 activity (Cornec-Le Gall et al., 2019). This imbalance leads to cyst-lining cells exhibiting overproduction of cyclic AMP (cAMP) levels, promoting cyst formation by stimulating cellular proliferation and cyst enlargement by activating protein kinase A and the Ras/Raf/ERK pathway (Cornec-Le Gall et al., 2019). This also leads to increased extracellular matrix deposition and fluid secretion through the cystic fibrosis transmembrane conductance regulator (CFTR), further exacerbating cyst growth (Hanaoka and Guggino, 2000).

However, PC1 and PC2 are known to regulate multiple more signaling pathways in ADPKD, and their dysfunction drives cystogenesis through various mechanisms (Figure 1); (Malek et al., 2019; Carracedo et al., 2008; Liu et al., 2018). The PI3K (phosphatidylinositol-4,5-bisphosphate 3-kinase)/Akt pathway is activated in ADPKD, modulating apoptosis, metabolism, and cell survival, while also altering calcium homeostasis by recruiting PC2 to the plasma membrane to function as a calcium influx channel (Malek et al., 2019; Carracedo et al., 2008; Liu et al., 2018). PI3K/Akt dysregulation contributes to hyperproliferation by altering the G2-M cell cycle checkpoint, leading to unchecked cell division in cystic epithelial cells (Fragiadaki, 2022). Disruptions in cellular metabolism are also implicated in ADPKD, with studies showing that cystic kidney cells exhibit altered glucose and lipid metabolism, similar to metabolic shifts observed in cancer cells (Malek et al., 2019; Steidl et al., 2023; Clerici and Boletta, 2025). Increased glycolysis and mitochondrial dysfunction create an environment favoring cystic cell survival and growth (Malek et al., 2019). Activation of AMP-activated protein kinase (AMPK), a key metabolic regulator, has been proposed as a therapeutic target to counteract these metabolic disturbances (Takiar et al., 2011). Metformin, an AMPK activator, has shown promise in reducing cystic growth in preclinical models (Takiar et al., 2011).

**FIGURE 1:**
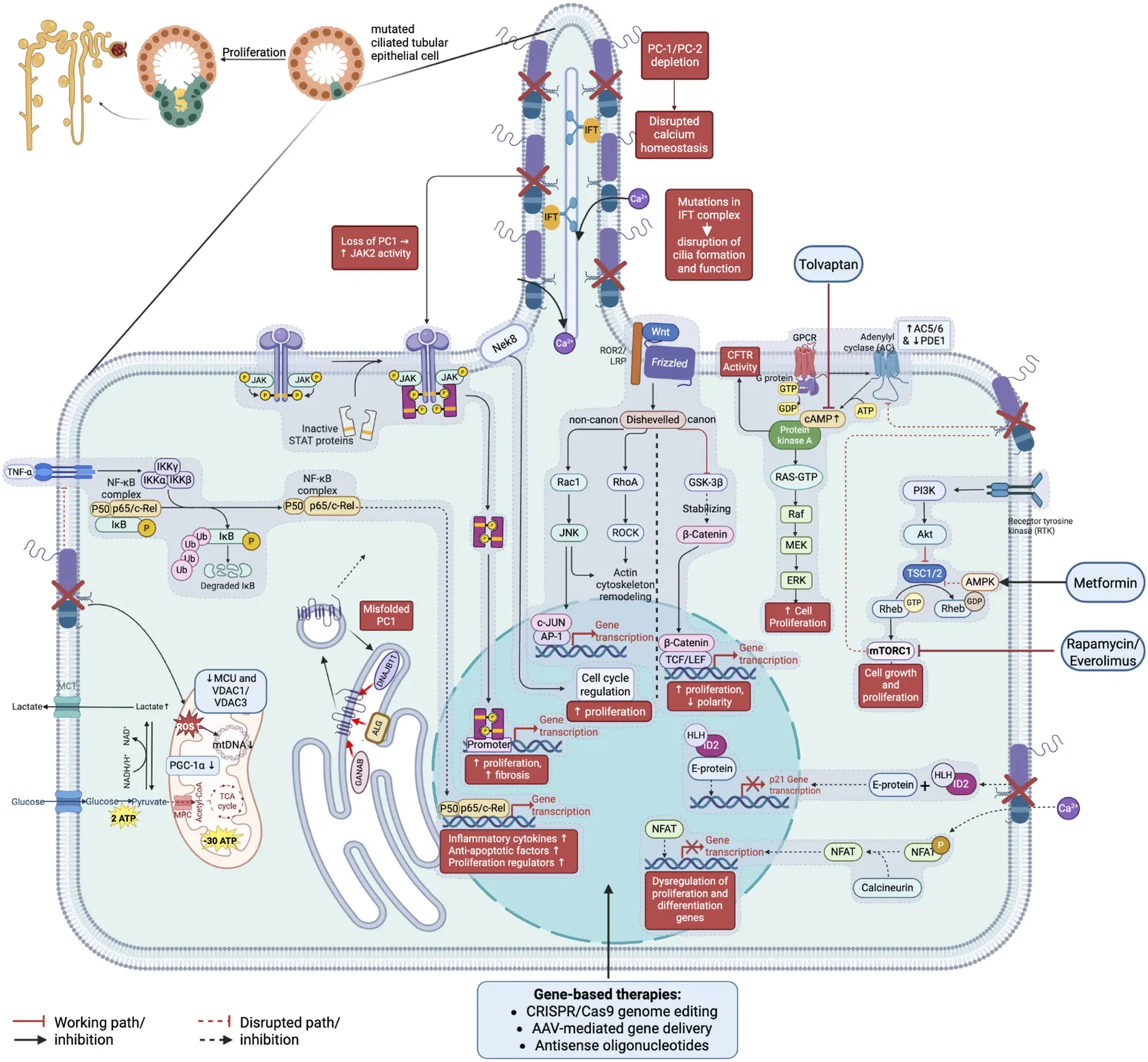
ADPKD cystopathogenesis pathways and related treatments. The figure depicts major molecular and cellular mechanisms involved in ADPKD pathogenesis. Shown are: loss of PC1/PC2 function and disrupted calcium homeostasis; mutations in the intraflagellar transport (IFT) complex affecting cilia formation; altered JAK/STAT, NF-κB, Wnt/β-catenin, and mTOR signaling; abnormal Ras/MAPK and PI3K/Akt activation; dysregulated cAMP signaling; ER stress due to misfolded PC1; and mitochondrial dysfunction with altered glycolysis and lactate production. Pharmacological interventions illustrated include Tolvaptan (V2 receptor blockade), Metformin (AMPK activation), Rapamycin/Everolimus (mTOR inhibition), and modulators of PDE and calcineurin pathways. Emerging gene-based therapies (CRISPR/Cas9, AAV-mediated delivery, antisense oligonucleotides) are shown at the bottom. Solid red lines denote active signaling, dashed red lines indicate disrupted or inhibited pathways.

The JAK-STAT pathway is influenced by polycystins, with PC1 activating JAK2 to phosphorylate STAT proteins, particularly STAT1 and STAT3, which then regulate gene transcription (Qin et al., 2010). This pathway is involved in controlling cell cycle arrest through p21 activation (Bhunia et al., 2002), while STAT6 activation contributes to cystic cell proliferation (Low et al., 2006). Additionally, the study highlights STAT5 upregulation in cystic epithelial cells, linking it not only to proliferation but also to fibrotic remodeling in ADPKD (Fragiadaki, 2022). This suggests that STAT5 activation contributes to extracellular matrix deposition and tissue stiffening, exacerbating kidney dysfunction (Fragiadaki, 2022). Furthermore, chronic inflammation plays a significant role in ADPKD progression, with the JAK-STAT and nuclear factor-kappa B (NF-κB) pathways being upregulated in cystic kidneys (Malek et al., 2019; Karihaloo, 2015). This leads to increased production of pro-inflammatory cytokines and fibrosis, as inflammatory cells infiltrate the cystic microenvironment, exacerbating epithelial proliferation and extracellular matrix remodeling (Malek et al., 2019; Karihaloo, 2015). Targeting these pathways with JAK-STAT blockers and COX-2 inhibitors has been explored as a potential therapeutic approach to slow cyst expansion (Malek et al., 2019).

The Wnt signaling pathway, a key regulator of embryonic development and tissue homeostasis, is dysregulated in ADPKD, contributing to cystic cell proliferation and loss of polarity (Steinhart and Angers, 2018). While canonical Wnt signaling stabilizes β-catenin to drive transcription, PC1 interferes with β-catenin’s interaction with TCF, thereby influencing cystogenesis (Clevers, 2006). Non-canonical Wnt signaling, which regulates intracellular calcium levels, is also affected, as Wnt ligands interact with PC1 and PC2, altering cellular polarity and motility (Sugimura and Li, 2010).

While *PKD1* and *PKD2* mutations both disrupt polycystin-mediated signaling, leading to altered calcium homeostasis, increased cAMP levels, and cystic expansion, their molecular effects and clinical impact differ significantly. *PKD1* and *PKD2* mutations differ not only in their prevalence but also in varying severity of disease progression and age of ESKD onset (Gall et al., 2013). Understanding the distinct characteristics of these mutations is essential for unraveling the variability in ADPKD progression and guiding precision medicine approaches.

*PKD1*, located on chromosome 16 (16p13.3), encodes PC1, a large transmembrane protein with a molecular weight of 500 kD and consists of 11 transmembrane domains (Malek et al., 2019). Its structural similarities to G-protein coupled receptors (GPCRs) suggest its involvement in complex signaling pathways (Malek et al., 2019). PC1 is primarily localized to the plasma membrane of primary cilia, as well as in cell junctions such as desmosomal junctions (Malek et al., 2019). Due to its diverse localization, PC1 plays a crucial role in cell–cell and cell–matrix adhesion, in addition to its functions in ciliary signaling (The European Polycystic Kidney Disease C, 1994; Fedeles et al., 2014). As mentioned above, it is the more common of the two ADPKD mutations. It is associated with a more severe disease course, leading to an earlier onset of ESKD compared to *PKD2* (Agborbesong et al., 2022), with average ages of 58.0 and 74.8 years, respectively (Gall et al., 2013). The more severe phenotype associated with *PKD1* mutations likely reflects their high penetrance and widespread loss of PC1 function, which promotes extensive cystic involvement of renal tissue (Mantovani et al., 2020). The clinical impact also varies by mutation type, with patients carrying truncating *PKD1* variants reaching ESKD approximately 9–13 years earlier than those with non-truncating *PKD1* or *PKD2* mutations (Mantovani et al., 2020). A challenge related to the genetic analysis of it, is the precense of six pseudogenes (*PKD1P1* - *PKD1P6)* located on chromosome 16, which complicates its diagnosis compared to other causative genes (Mantovani et al., 2020). While the pathways described above are a shared burden of *PKD1* and *PKD2*, some might be unique to expression of each. The TSC-mTOR (tuberous sclerosis complex-mammalian target of rapamycin) pathway is negatively regulated by PC1, which interacts with tuberin (TSC2) to maintain its inhibitory role on mTOR (Boletta, 2009). This pathway controls cell growth, proliferation, apoptosis, and cytoskeletal regulation, but dysfunction leads to abnormal cystogenesis (Boletta, 2009). PC1 also activates G-protein signaling, leading to AP-1 transcription factor activation via JNK and protein kinase C (PKC), further influencing cellular differentiation, proliferation, and apoptosis (Boletta, 2009). Additionally, the calcineurin/nuclear factor of activated T-cells (NFAT) pathway is linked to PC1 activity, mediating calcium-dependent transcriptional regulation, though its precise role in ADPKD remains under investigation (Reiterová and Tesař, 2022; Puri et al., 2004).

*PKD2*, located on chromosome 4 (4q21) encodes PC2, a calcium-selective channel belonging to the transient receptor potential (TRP) channel family (Malek et al., 2019). It has six transmembrane domains and a molecular weight of 110 kD (Malek et al., 2019). PC2 is found in multiple subcellular locations, including the plasma membrane, endoplasmic reticulum (ER), and primary cilia (Busch et al., 2017). Beyond its role in calcium homeostasis, PC2 is also integral to centrosome structure and mitotic spindle organization. Dysfunction of PC2 results in decreased intracellular calcium levels, which contributes to cystogenesis in ADPKD (Grieben et al., 2016; Mochizuki et al., 1996; Yang and Ehrlich, 2016). It has fewer occurrences and milder effect, which might be due to *PKD2* having variable expressivity. However, the complete depletion of PC2 due to *PKD2* knockout (KO) was shown to have serious repercussions via RNA sequencing, altering nearly 900 genes linked to cilia function, Wnt signaling, and MAPK pathways (Jung et al., 2023). This supports a PC2-dependent cilia-to-nucleus signaling axis in cyst initiation. Disrupted Wnt signaling was marked by upregulated *DVL2* (Sharma et al., 2018) and downregulated *DCDC2A* (Schueler et al., 2015), indicating early involvement in cyst formation. MAPK activation in *PKD2*-KO cells led to increased p54 JNK and ERK, with immunoblot validation in ADPKD mouse models confirming their role in cystogenesis (Jung et al., 2023). GLIS3, a key transcription factor in ciliogenesis, was significantly downregulated, potentially activating Wnt signaling and driving uncontrolled cell proliferation, further linking PC2 loss to cystic expansion (Jung et al., 2023). There also other pathways that are specifically connected to the expression of *PKD2.* The Id (inhibitor of DNA binding) pathway is a regulatory mechanism that modulates transcriptional control through interactions between PC2 and Id2, preventing its binding to E-protein transcription factors (Li et al., 2005).

Beyond the canonical signaling pathways, mitochondrial dysfunction represents another critical axis in ADPKD pathogenesis. Polycystin deficiency shifts metabolism from oxidative phosphorylation (OXPHOS) to glycolysis (Warburg-like effect) (Saravanabavan and Rangan, 2021), causing mitochondrial fragmentation, reduced membrane potential, and the accumulation of reactive oxygen species (ROS), that drive cyst expansion (Ishimoto et al., 2017). ADPKD models exhibit structural mitochondrial abnormalities including damaged cristae, reduced mitochondrial DNA (mtDNA) copy numbers, and mutations in ND4 and COX2 that impair electron transport (Saravanabavan and Rangan, 2021; Ishimoto et al., 2017). A central feature is disrupted calcium homeostasis: *PKD1/PKD2* loss reduces expression of key calcium transport proteins (MCU and VDAC1/VDAC3) (Yanda et al., 2022), impairing mitochondrial Ca^2+^ uptake, which decreases ATP production from the normal 30 ATP to only 2 ATP per glucose molecule (Ishimoto et al., 2017; Yanda et al., 2022). The resulting bioenergetic stress and downregulation of PGC-1α (a mitochondrial biogenesis regulator) further compromise mitochondrial function (Ishimoto et al., 2017). These changes, along with chronic DNA damage response activation, promote cystic epithelial cell proliferation and contribute to ADPKD progression (Zhang JQJ. et al., 2021).

### 2.3 Rare ADPKD-Associated genes

While the dysregulation of signaling and metabolic pathways explains much of ADPKD pathophysiology, recent advances in genomic sequencing have revealed additional genetic contributors that expand the spectrum of the disease beyond *PKD1* and *PKD2*. Mutations in genes such as *GANAB, DNAJB11, ALG9, IFT140, ALG5,* and *NEK8* (Table 1) have been implicated in atypical or milder ADPKD phenotypes (Hanaoka and Guggino, 2000; Malek et al., 2019). *GANAB*, also sometimes called *PKD3* (Porath et al., 2016), encodes the alpha subunit of glucosidase II, which forms a functional holoenzyme within the ER. Its dysfunction has been linked to defects in the maturation and localization of PC1. (Porath et al., 2016; Cordido et al., 2017). Notably, unlike *PKD1* and *PKD2*, *GANAB* is rarely linked to ESKD but is also implicated in autosomal dominant polycystic liver disease (ADPLD), a condition related to ADPKD that primarily affects the liver while causing minimal kidney cyst formation (Senum et al., 2022).

Another gene implicated in ADPKD is *DNAJB11*, which encodes ERdj3, a co- factor of the chaperone protein BiP, involved in ER-associated protein processing. This protein plays a crucial role in ensuring the proper trafficking of PC1 (Cornec-Le Gall et al., 2018; Senum et al., 2022). *DNAJB11*-related nephropathy is characterized by the formation of small kidney cysts and fibrosis, eventually leading to ESKD later in life.

In contrast, the *ALG9* phenotype presents with moderate cystic kidney disease, with occasional progression to ESKD (Besse et al., 2019). *ALG9* encodes the *ALG9* alpha-1,2-mannosyltransferase, and along with *DNAJB11*, these proteins are involved in glycosylation, folding, quality control, and trafficking of membrane and secreted proteins within the ER (Besse et al., 2019). The loss or reduction of these ER proteostasis proteins particularly affects the processing of PC1, a large, glycosylated membrane protein, leading to impaired function and contributing to cystogenesis (Senum et al., 2022).

*IFT140*, a core component of the intraflagellar transport complex A (IFT-A), was recently identified as an ADPKD driver (Senum et al., 2022). Normally responsible for retrograde ciliary trafficking and membrane protein entry, its haploinsufficiency likely causes cyst formation by disrupting ciliary structure and reducing polycystin trafficking to cilia (Senum et al., 2022). Studies found *IFT140* variants in 1.9% of unscreened ADPKD families and 2.1% of cystic kidney disease cases in Genomics England 100K. In UK Biobank, *IFT140* pathogenic variants were the third most common cause after *PKD1* and *PKD2*, exceeding *GANAB* (Senum et al., 2022). The *IFT140*-related ADPKD phenotype is notably milder than classic ADPKD.

Monoallelic *ALG5* variants cause atypical ADPKD with late-onset disease (after age 60) (Lemoine et al., 2022). *ALG5* encodes an ER enzyme essential for N-glycosylation that affects protein maturation. The phenotype resembles *DNAJB11*-associated disease: non-enlarged kidneys with small cysts, progressive interstitial fibrosis, and ESKD in elderly patients (62–91 years) (Lemoine et al., 2022). These cases are likely under-diagnosed due to their mild presentation and late onset (Lemoine et al., 2022).

*NEK8* heterozygous variants in the kinase domain (primarily p. Arg45Trp, also p. Ile150Met and p. Lys157Gln) cause ADPKD through a dominant-negative mechanism (Claus et al., 2023). Unlike previously known biallelic *NEK8* mutations causing multiorgan disease, these heterozygous variants primarily cause renal pathogenesis (Claus et al., 2023). Functional studies revealed normal *NEK8* localization to cilia but disrupted PC2 trafficking (especially with p. Arg45Trp), reduced kinase activity, and increased DNA damage signaling. The clinical presentation varies by variant: p. Arg45Trp and p. Lys157Gln cause severe early-onset disease with enlarged kidneys, hypertension, and early kidney failure, while p. Ile150Met and mosaic cases show a milder phenotype with later onset (Claus et al., 2023).

Altought he rare mutaions described above do explain some of the non-*PKD1/2* related diagnosis, there are some cases where even this line of explanation is not applicable. It is speculated that a subset of the remaining cases arises post-zygotically due to somatic mosaicism, meaning that only some of the cells in the body display the mutation. In the NGS (next-generation sequencing) study of 20 families exhibiting *de novo* cases, it was found that mosaicism accounts for about 1% of all ADPKD cases and approximately 10% of genetically unresolved cases (Hopp et al., 2020). All identified mosaic cases involved *PKD1* mutations, with the majority being insertions or deletions (14 out of 20 cases) (Hopp et al., 2020). Patients with mosaic mutations displayed milder disease progression compared to those with complete *PKD1* mutations (Hopp et al., 2020). Their kidney function was relatively preserved, as indicated by higher estimated glomerular filtration rates (eGFR) and smaller total kidney volumes (TKV) than typically seen in full *PKD1* cases (Hopp et al., 2020). The study also found that mosaic mutations were transmitted to the next generation in five families, who exhibited both somatic and germline inheritance patterns, while the remaining fifteen cases were purely somatic, meaning they occurred post-zygotically and were not passed on (9). Notably, there were parents with mosaic pattern who had offspring inheriting fully penetrated *PKD1* mutation and developing a much more severe ADPKD phenotype (Hopp et al., 2020). Disease severity varied based on the proportion of affected cells in everyone, but there was no direct correlation between the level of mosaicism in blood samples and kidney phenotype. In some cases, unilateral or asymmetric kidney disease was observed, reinforcing the idea that mosaic mutations lead to a localized distribution of cyst formation, contributing to milder disease (Hopp et al., 2020).

### 2.4 Current methods for genetic diagnosis of ADPKD

According to KDIGO 2025 guidelines, the testing is most valuable in the cases of patients with few kidney cysts, variable intrafamilial disease severity, very-early-onset ADPKD, when imaging and kidney function are discordant, patients with negative family history, young (aged <30 years) living-related kidney donors at risk of ADPKD, and family planning with preimplantation diagnosis (Torres et al., 2025). A PKD or nephrology NGS panel is recommended as the most targeted and cost-effective method to genetically screen people with suspected ADPKD (Mantovani et al., 2020). Sanger sequencing of the target gene is usually sufficient in families with previously identified mutations (Mantovani et al., 2020). Whole-genome sequencing (WGS) may be used if there are atypical features or reasons to suspect variants other than *PKD1* and *PKD2* (Mallawaarachchi et al., 2021).

However, with the evolution of the genetic diagnosis of ADPKD in recent years, NGS has emerged as the predominant approach. As was mentioned in the earlier chapter, the technical challenges of diagnosing ADPKD stem primarily from the *PKD1* gene structure, which includes six pseudogenes with over 98% sequence homology to the first 33 exons, extensive allelic heterogeneity with over 1,500 known pathogenic variants, and the large size and high GC content of *PKD1* (Cornec-Le Gall et al., 2018). Current gold-standard genetic testing typically employs one of two NGS-based strategies: PCR-based methods using long-range PCR (LR-PCR) to specifically amplify *PKD1* while excluding pseudogenes followed by NGS, or DNA capture-based methods that use sequence capture approaches to enrich PKD genes before sequencing (Lanktree et al., 2019). Complementary techniques remain essential, including Multiplex Ligation-dependent Probe Amplification (MLPA) to detect large deletions or duplications that account for 1–3% of cases, and Sanger sequencing to confirm variants detected by NGS.

Modern testing panels now incorporate not only the primary causative genes *PKD1* and *PKD2*, but also rare causative genes including *GANAB, DNAJB11, ALG9, ALG5, IFT140,* and *NEK8* (Ali et al., 2019). A recent large-scale cohort of Italian patients demonstrated that targeted NGS panels achieved a high diagnostic yield, identifying pathogenic or likely pathogenic variants in approximately 78% of cases (Nigro et al., 2023). Importantly their analysis also underscored persistent limitations of current diagnostic workflow. Particularly the challenges in variant interpretation and detection of complex structural rearrangements (Nigro et al., 2023). These findings reinforce the clinical relevance of NGS-based testing while emphasizing the need for complementary methods and careful variant classification (Nigro et al., 2023). Variant classification adheres to American College of Medical Genetics and Genomics (ACMG) guidelines, though up to 26% of identified nucleotide variants remain difficult to classify functionally (Song et al., 2017). The comprehensive NGS-based approach has significantly reduced both costs and turnaround times compared to traditional methods, expanding access to genetic testing and enabling its integration into routine clinical care for ADPKD patients (Mantovani et al., 2020; Mallawaarachchi et al., 2021).

### 2.5 Long-read sequencing in ADPKD diagnostics

Despite the advances made with standard NGS approaches, emerging long-read sequencing (LRS) technologies represent the cutting edge of ADPKD genetic diagnostics, addressing persistent limitations in conventional testing methods. Traditional approaches using LR-PCR followed by Sanger sequencing has been the gold standard for many years. LR-PCR enables the specific amplification of the *PKD1* gene from among its six highly homologous pseudogenes, generating large DNA fragments (2–6 kb) that cover multiple exons simultaneously (Tan et al., 2012). However, these methods are labor-intensive, time-consuming, and expensive (Tan et al., 2012), which is being solved by recent technological advances in LRS. The NGS method was shown to be superior to Sanger sequencing for detecting PKD gene mutations, achieving high sensitivity and improved gene coverage. Direct sequencing of LR-PCR fragments allows for comprehensive coverage of the entire *PKD1* gene, circumventing the need for nested PCR of individual exons (Tan et al., 2012). This approach substantially reduced the number of PCRs by 80%, decreasing both cost and the potential for allele dropout during amplification. These characteristics suggest that these advanced approaches would be an appropriate new standard for clinical genetic testing of ADPKD.

LRS technologies, such as those from Oxford Nanopore and PacBio, can generate reads spanning several kilobases to tens of kilobases, enabling the accurate resolution of the *PKD1* pseudogene regions (Xu et al., 2024). This represents a significant advantage over short-read NGS, which struggles with sequence homology in *PKD1’s* duplicated regions (Mallawaarachchi et al., 2021). A recent study demonstrated that a targeted LRS approach identified pathogenic *PKD1* variants in patients who remained undiagnosed after short-read sequencing, increasing diagnostic yield by up to 25% in previously unsolved cases (Sun et al., 2025).

LRS offers several distinct benefits for ADPKD genetic testing, such as improved detection of complex structural variants, better resolution of pseudogene regions, detection of deep intronic variants, and direct phasing of variants. Firstly, LRS can identify large deletions, duplications, and rearrangements that short-read technologies often miss (Sun et al., 2025). This is particularly valuable for *PKD1*, where pathogenic variants include these structural alterations. Secondly, the ability to span entire pseudogene regions with single reads eliminates alignment ambiguities that plague short-read sequencing, enabling more confident variant calling in the duplicated regions of *PKD1* (Ali et al., 2019). Thirdly, LRS can identify pathogenic variants in non-coding regions, such as deep intronic mutations affecting splicing, which has revealed a new class of disease-causing variants in previously unsolved cases (Hort et al., 2023). Lastly, LRS enables direct determination of variant phase (whether variants are on the same or different chromosomes), which is particularly valuable in cases with multiple variants of uncertain significance (Peng et al., 2023).

One study case has also shown how LRS is able to uncover unusual cases over looked by more widespread NGS methods (Qiu et al., 2024). In a family with ADPKD, researchers discovered that what initially appeared to be a *PKD1* exon deletion using conventional genetic testing (WES and MLPA) was actually a gene conversion event where a 282 bp segment of *PKD1* had been replaced by pseudogene sequence (Qiu et al., 2024). This conversion introduced seven variants, including a pathogenic c.7288C>T that creates a premature stop codon (Qiu et al., 2024). The findings highlight the limitations of standard genetic testing for complex regions like *PKD1* and demonstrate the superior accuracy of LRS technology for diagnosing ADPKD (Qiu et al., 2024).

Most laboratories are now implementing hybrid approaches that leverage the strengths of both technologies. For example, the “Comprehensive Analysis of ADPKD” (CAPKD) assay combines multiplex LR-PCR with LRS to achieve highly specific analysis of *PKD1* with minimal pseudogene interference (Xu et al., 2024). Similarly, WGS with targeted analysis provides comprehensive coverage of all known ADPKD genes, achieving diagnostic rates of over 80% in patients with typical ADPKD presentation (Mallawaarachchi et al., 2021). These technological advances are particularly valuable for cases requiring precise genetic diagnosis, such as genetically unresolved families, evaluation of potential living-related kidney donors, preimplantation genetic testing, and cases with atypical presentation or early onset disease (Hort et al., 2023; Peng et al., 2023). As these technologies become more accessible and cost-effective, they are likely to become the new standard for ADPKD genetic testing, enabling more complete genetic characterization and better informed clinical management.

## 3 Epigenetic and post-translational modifications for disease prognosis

### 3.1 DNA methylation

Epigenetic modifications may also contribute to the rest of the ADPKD cases not caused by *PKD1* or *PKD2* mutations (Yan et al., 2025; Agborbesong et al., 2022). Currently, there are a few known epigenetic mechanisms affecting the progression of ADPKD without altering the DNA (Figure 2); (Agborbesong et al., 2022). Most studied mechanisms are those of methylation and histone modifications. The former is regulated by *DNMT* (DN methyl transferase) enzyme that attaches methyl groups to the nucleic acid chains (Agborbesong et al., 2022). Depending on where in the DNA sequence methyl group is applied it leads to either repression or overexpression (Agborbesong et al., 2022). ADPKD is associated with hypermethylation of *PKD1* gene body, which has been reported to silence expression and lead to consequent downregulation of PC1 (Steinhart and Angers, 2018; Woo et al., 2013). In addition to the *PKD1* gene body, global methylation profiling has revealed that 91% of methylation changes in ADPKD kidneys are hypermethylation events (11,999 fragments hypermethylated vs. only 1,228 hypomethylated), indicating a systemic shift in epigenetic regulation (Woo et al., 2013). Notably, *DNMT1* expression is markedly elevated in cystic epithelial cells, further promoting aberrant silencing of regulatory genes beyond *PKD1* (Yan et al., 2025; Zhou et al., 2024). Therapeutically, targeted inhibition of *DNMT1* in ADPKD models has reduced cyst burden and extended lifespan, highlighting a critical functional role for methylation machinery in disease progression (Yan et al., 2025; Agborbesong et al., 2022; Zhou et al., 2024). Another study found that *PKD1* promoter methylation was significantly lower in ADPKD patients, with an average of 18.9% compared to 62.5% in healthy controls, with a strong negative correlation between methylation and PKD1 expression (r = −0.53, p = 0.0162 in patients; r = −0.63, p = 0.0031 in controls) (Hajirezaei et al., 2020). *PKD1* gene expression was higher in ADPKD patients, and a negative correlation was observed between *PKD1* promoter methylation and its expression, indicating that decreased methylation may upregulate *PKD1* transcription (Hajirezaei et al., 2020). Age-related differences in *PKD1* promoter methylation were also identified, with younger ADPKD patients showing higher methylation levels compared to older patients, suggesting that epigenetic changes may shift over time (Hajirezaei et al., 2020). These findings contrast with previous studies that focused on gene-body methylation rather than promoter methylation, providing new insights into epigenetic regulation in ADPKD (Hajirezaei et al., 2020). The results indicate that DNA methylation may play a role in disease progression and could serve as a potential biomarker or therapeutic target (Hajirezaei et al., 2020). However, the study acknowledges that blood-derived methylation patterns may not fully reflect kidney tissue-specific changes, and further research is needed to explore the functional consequences of *PKD1* promoter methylation in cyst development (Hajirezaei et al., 2020). Interestingly, recent data suggest that *PKD1* promoter hypomethylation might not be a compensatory mechanism but part of a broader epigenetic dysregulation (Yan et al., 2025, Ali et al., 2024, Bowden et al., 2020). For example, *MUPCDH* (MUC1-like protocadherin gene), another renal gene silenced via promoter hypermethylation, is reactivated by demethylating agents, implicating a recurring motif of gene repression via CpG island hypermethylation across key loci in ADPKD (Mi Woo et al., 2015; Yan et al., 2025). This reinforces the idea that gene-specific hypermethylation events (e.g., *PKD1, MUPCDH*, miR-192/194) may function as strong pathogenic drivers, while global shifts act more as background reprogramming (Mi Woo et al., 2015; Bowden et al., 2018; Kim et al., 2019; Yan et al., 2025).

**FIGURE 2:**
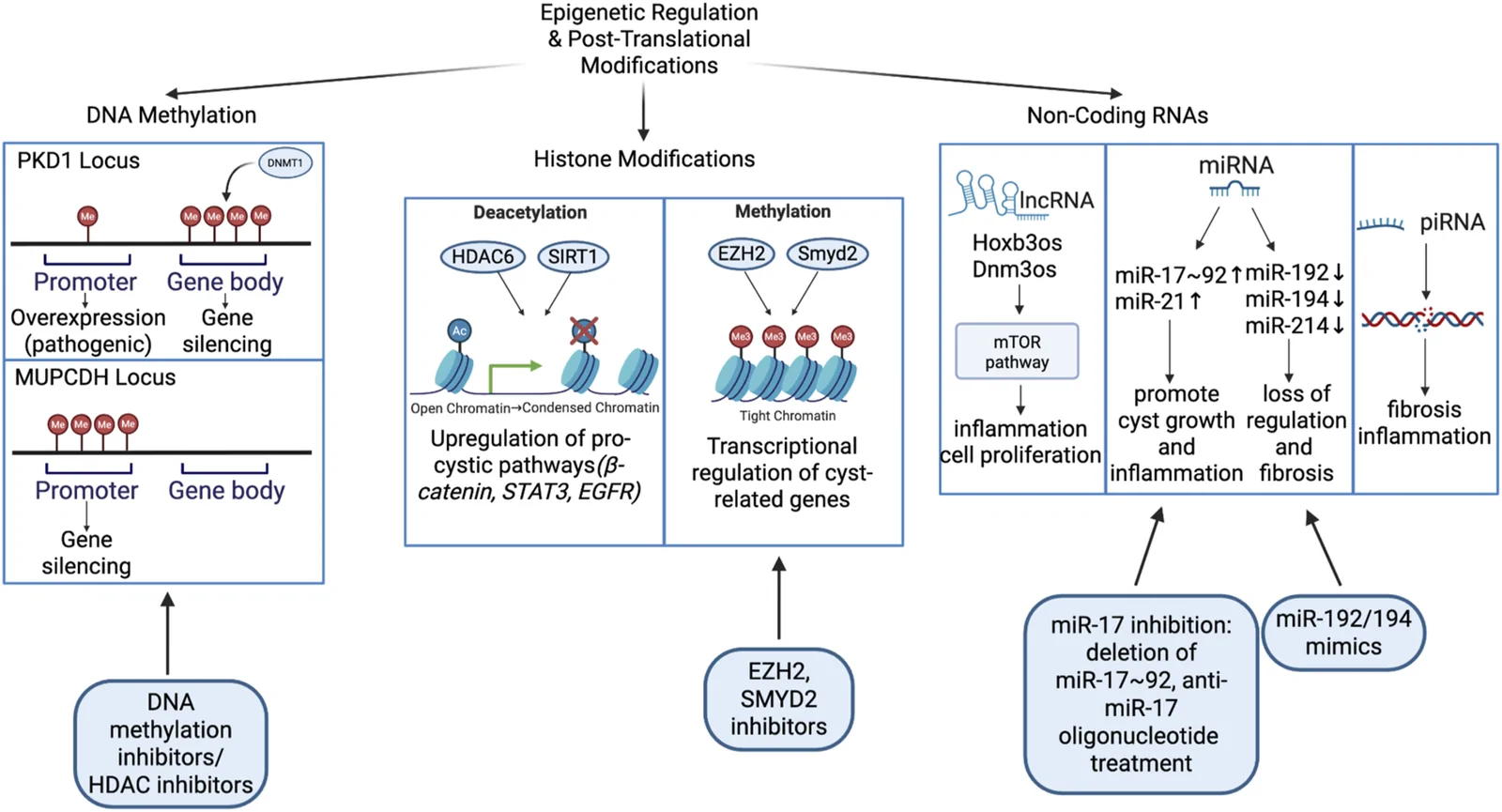
Epigenetic regulation and Post-translational modifofcations in ADPKD. Alterations in DNA methylation, histone modifications, and non-coding RNA activity contribute to cystogenesis. Hypermethylation at the *PKD1* and *MUPCDH* loci leads to abnormal gene expression and silencing. Histone deacetylation (HDAC6, SIRT1) and methylation (EZH2, SMYD2) promote chromatin condensation and transcriptional activation of cyst-promoting pathways (*β-catenin*, *STAT3*, *EGFR*). Non-coding RNAs—including lncRNAs (*Hoxb3os*, *Dnm3os*) and miRNAs (*miR-17∼92*, *miR-21*, *miR-192/194/214*)—modulate mTOR signaling, inflammation, and fibrosis, while piRNAs contribute to fibrotic regulation. Potential therapeutic approaches include DNA methylation and HDAC inhibitors, EZH2/SMYD2 inhibitors, anti-miR-17 oligonucleotides, and miR-192/194 mimics.

ADPKD cysts exhibit widespread epigenetic alterations, with global DNA methylation levels significantly reduced compared to non-ADPKD kidneys, indicating epigenetic reprogramming in disease progression (Bowden et al., 2020). On average, ADPKD genomes are 3–4% hypomethylated relative to controls, consistent with tumor-like epigenetic drift (Yan et al., 2025). While whole kidney tissue shows some degree of hypomethylation, individual cysts are more profoundly affected, suggesting that methylation changes occur at the cyst level rather than uniformly across the kidney (Bowden et al., 2020). Single-cyst analysis identified 96 common differentially methylated fragments (DMFs) enriched in organ development, cell adhesion, and Notch signaling pathways, all of which contribute to cyst formation and kidney dysfunction (Bowden et al., 2020). In fact, depending on the cyst, between 474 and 6,087 DMFs were detected, with 6,727 inter-cyst variants (ICVs) representing approximately 15% of the analyzed genome, underscoring how each cyst follows its own molecular trajectory (Bowden et al., 2020). Chromosomes 4 and 17 exhibited notable methylation variability, although its precise impact remains unclear (Bowden et al., 2020). 2415 genes displayed inter-cyst methylation differences, many linked to embryonic development and morphogenesis, reinforcing the idea that cystic epithelial cells undergo reprogramming akin to early kidney development (Bowden et al., 2020). Further analysis found that 837 DMFs also exhibited inter-cyst variability, confirming that methylation changes in cystic tissue differ significantly between cysts (Bowden et al., 2020). This suggests each cyst may develop through independent molecular mechanisms, contributing to disease heterogeneity. Similar to epigenetic reprogramming in cancer and other progressive diseases, these stochastic methylation changes may influence cyst expansion and disease severity, offering new insights into ADPKD pathogenesis (Bowden et al., 2020).

### 3.2 Non-coding RNAs and histone modification

Beyond methylation, epigenetic mechanisms play a broader role in ADPKD progression, influencing gene expression through histone modifications and non-coding RNA regulation. Hypermethylation of genes outside *PKD1*, such as *MUPCDH* gene and miRNAs like miR-192 and miR-194 (Hajirezaei et al., 2020; Mi Woo et al., 2015) exacerbates cyst growth, while demethylating agents can potentially reverse these effects (Yan et al., 2025). Histone modifications, including methylation and acetylation, regulate chromatin accessibility and gene transcription, impacting cyst development. Key histone-modifying enzymes, such as SMYD2 and EZH2, are critically dysregulated in ADPKD (Xu et al., 2022). For example, SMYD2, a histone lysine methyltransferase upregulated in ADPKD cyst-lining cells (Xu et al., 2022), methylates non-histone proteins like p53, STAT3, and NF-κB p65, therefor enhancing pro-proliferative and pro-survival signaling in cyst epithelia (Xu et al., 2022). EZH2, on the other hand, is the catalytic subunit of the Polycomb repressive complex 2, which tri-methylates H3K27 (Xu et al., 2022). It is markedly overexpressed in cystic kidneys and appears to promote cell cycle progression, likely via the Rb/E2F pathway​ (Xu et al., 2022). Together, these findings indicate that dysregulated histone methyltransferases directly influence cyst growth and disease progression in ADPKD. Additionally, histone deacetylases (HDAC6 and SIRT1) influence cyst growth through pathways like EGFR signaling and β-catenin translocation, making them viable drug targets (Yan et al., 2025). HDAC6 causes EGFR mislocalization to the apical membrane, triggering ERK1/2 phosphorylation and disrupting Ca^2+^/cAMP signaling pathways that increase cyst fluid accumulation (Yan et al., 2025).

Non-coding RNAs, including miRNAs and long non-coding RNAs (lncRNAs), modulate key pathways in ADPKD. Upregulated miRNAs, such as miR-17–92 and miR-21, promote cyst growth and inflammation, while others, like miR-199a-5p, promotes cyst expansion through suppression of the CDKN1C/p57 pathway (Yan et al., 2025; Zhou and Li, 2021). Downregulated miRNAs, including miR-192, miR-194, and miR-214, are associated with fibrosis and loss of cystic cell regulation, with miR-214 specifically linked to TLR4-mediated inflammatory signaling (Yan et al., 2025; Zhou and Li, 2021). In addition to miRNAs, lncRNAs such as Hoxb3os and Dnm3os contribute to ADPKD pathogenesis by regulating mTOR signaling and inflammation, often interacting with miRNAs to modulate cyst growth (Zhou and Li, 2021). Experimental downregulation of Hoxb3os, for example, increased renal epithelial cell proliferation by approximately 50–60%, underscoring its role as a growth suppressor (Yan et al., 2025). Recent findings also emphasize the role of miRNA-mediated regulation in modulating key signaling pathways such as PI3K/Akt and STAT5 (Fragiadaki, 2022). Dysregulated miRNAs contribute to aberrant activation of these pathways, reinforcing the interplay between genetic mutations and epigenetic factors in ADPKD progression. This suggests that targeting specific miRNAs could serve as a novel therapeutic strategy to disrupt pathogenic signaling and slow disease progression.

### 3.3 Role of piRNAs in ADPKD

Piwi-interacting RNAs (piRNAs) a unique group of small non-coding RNAs measuring 24–32 nucleotides, are increasingly recognized for their role in ADPKD pathogenesis. Originally known for their functions in germ cells, piRNAs contribute to cyst development and disease progression via epigenetic regulatory mechanisms (Ali et al., 2024; Ozata et al., 2018). Unlike miRNAs, which are processed from double-stranded precursors by Dicer and associate with AGO proteins, piRNAs are derived from long single-stranded precursors and interact with PIWI-clade Argonaute proteins (Ozata et al., 2018). They play crucial roles in silencing transposable elements, guiding epigenetic modifications (e.g., DNA methylation), and preserving genome stability (Ozata et al., 2018). piRNAs are typically 2′-O-methylated at the 3′ end, enhancing their stability, and are less conserved across species than miRNAs (Ozata et al., 2018). Whereas miRNAs predominantly modulate gene expression in somatic cells by targeting mRNAs, piRNAs operate mainly in the germline, where they safeguard genomic integrity through transposon silencing, chromatin remodeling, and other epigenetic mechanisms (Ali et al., 2024, Ozata et al., 2018). Twenty-three piRNAs were found to be dysregulated, with piR_020497, piR_016271, and piR_020496 showing significant alterations (Ali et al., 2024). Their role in epigenetic modifications and genomic stability may contribute to aberrant cell proliferation and loss of tubular architecture. Since piRNAs regulate transposable elements, their dysregulation in ADPKD may lead to genomic instability, a feature shared with other progressive kidney diseases and cancers. Additionally, piRNAs have been linked to fibrosis and inflammatory responses, which are central to cystic kidney disease progression. While their exact function in ADPKD remains unclear, their involvement in DNA methylation, histone modifications, and chromatin remodeling suggests they could be key players in cystogenesis and potential therapeutic targets.

Functional network analysis of dysregulated miRNAs in urinary extracellular vesicles identified key driver genes involved in ADPKD progression (Ali et al., 2024). *FBRS* (Fibrosin) regulates extracellular matrix remodeling and fibrosis, with its dysregulation suggesting increased fibrotic activity, a hallmark of progressive kidney disease. *EDC3* (Enhancer of mRNA Decapping 3) plays a role in mRNA degradation, and its suppression may prolong pro-cystic and pro-inflammatory transcripts. *FMNL3* (Formin Like 3) is involved in cytoskeletal organization and epithelial polarity, with its dysregulation potentially contributing to loss of polarity in cyst-lining cells. *CTNNBIP1* (Catenin Beta Interacting Protein 1) inhibits Wnt/β-catenin signaling, and its downregulation may lead to unchecked cell proliferation in cystic cells. *KMT2A* (Lysine Methyltransferase 2A), a histone methyltransferase, is crucial for chromatin remodeling and gene activation, and its altered expression could enhance cyst formation through histone methylation changes (Ali et al., 2024). Additionally, a comprehensive miRNA profiling study identified miR-192, miR-194, and miR-30 family members as significantly downregulated in ADPKD urine exosomes, with their suppression correlating with disease severity and kidney enlargement. These miRNAs regulate key signaling pathways, including PI3K-Akt and Wnt, by targeting genes such as *PIK3R1* and *ANO1*, which are involved in cyst expansion.

Epigenetic mechanisms, including DNA methylation, histone modifications, and non-coding RNAs, play a significant role in ADPKD progression. Variability in methylation patterns, particularly inter-cyst differences, suggests that cysts may form through independent molecular mechanisms. Dysregulated miRNAs and piRNAs further contribute to cystogenesis by altering gene expression, fibrosis, and inflammation. Additionally, urinary miRNA profiling has emerged as a potential biomarker for disease severity and progression. These findings highlight epigenetics as a key factor in ADPKD pathogenesis, offering potential therapeutic targets. Nevertheless, an important limitation is the difficulty in establishing causality. A major problem with epigenetic and post translational modifications is that in some cases it is hard to differentiate between cause (Yan et al., 2025; Agborbesong et al., 2022) and correlations (Bowden et al., 2020; Ali et al., 2024). As in how do we know if a certain change observed in ADPKD was a contributing factor to it or happened because of it or other related symptoms.

## 4 Diagnostic approaches of ADPKD

### 4.1 Differential diagnosis of ADPKD from other cystic diseases

In the literature, various cystic kidney diseases such as simple renal cysts, acquired cystic kidney disease, and polycystic nephroma have been revealed so far and each of them should be carefully diagnosed with ADPKD. Patients with ADPKD often have a family history of this disease (75–90% having a parent with ADPKD (Cornec-Le Gall et al., 2019). However, among patients with no family history (the 10–25% who have no parent with ADPKD (Cornec-Le Gall et al., 2019), some 40% reportedly have no observed mutation in the *PKD1* or *PKD2* genes (Iliuta et al., 2017); this suggests that careful diagnosis is required to differentiate between ADPKD and other cystic kidney diseases (Table 2). The development of simple renal cysts, acquired cystic kidney disease, and polycystic nephroma cysts are usually unilateral but with age, the number of cysts increases and the frequency with which they are present in both kidneys increases (Eknoyan, 2009). Another landmark difference of ADPKD from the abovementioned cysts is inheritance while the above-mentioned cysts differ in the absence of family history, and failure to progress to ESKD (Cornec-Le Gall et al., 2019). In most cases, these cysts have no extrarenal complications, such as hepatic cysts or cerebral aneurysms which are common in ADPKD. Depending on the mutation occurrence (most aggressive is *PKD1* and *PKD2*), cystic growth may vary among the patients with ADPKD, and usually the onset is in adulthood and needs more careful investigation with instrumental, laboratory, and molecular genetic findings. Although genetic testing is the standard criterion for diagnosis of ADPKD, it is not the first-line diagnostic tool, owing to its high cost and lack of broad availability. Based on recent KDIGO 2025 clinical practice guidelines for the evaluation, management, and treatment of autosomal dominant polycystic kidney disease to diagnosis diagnose adults at risk or with a positive family history for ADPKD is an important combination of family history, relevant clinical examination findings, and imaging manifestations, and then genetic testing which are usually sufficient for diagnosis of ADPKD (Torres et al., 2025). All the patients with a familial history of ADPKD according to KDIGO guidelines recommended abdominal imaging by ultrasound to early detect ADPKD onset (Torres et al., 2025). For people with a positive family history of ADPKD aged 16–40 years, the cutoff of >10 cysts on magnetic resonance imaging (MRI) have been used to diagnose ADPKD and the cutoff of <5 cysts has been used to exclude ADPKD (Torres et al., 2025). These ultrasound and MRI criteria apply to only families with pathogenic variants of *PKD1* or *PKD2*, and not to those with pathogenic variants of minor genes. In a family with a known pathogenic variant, targeted screening for the specific variant is usually sufficient to diagnose or exclude ADPKD. Imaging, therefore, plays a key role in the diagnosis and initial assessment of ADPKD. Imaging biomarker quantification methods like total kidney volume are actively used in clinical settings. Here we discuss classical and advanced diagnostic approaches in ADPKD.

**TABLE 2:** Other polycystic diseases genes.

Genes	Associated diseases
*UMOD, MUC1, REN, SEC61A*	Autosomal dominant tubulointerstitial kidney disease
*PRKCSH, SEC63, GANAB*	Autosomal dominant polycystic liver disease (ADPLD)
*PKHD1, DZIP1L, CYS1, PKD1*	Autosomal recessive polycystic kidney disease (ARPKD)
*PMM2*	Hyperinsulinemic hypoglycemia and polycystic kidney disease (HIPKD)

### 4.2 Imaging biomarkers of ADPKD progression

Following the differential diagnostics of ADPKD from other cystic contions, imaging also plays a central role in both diagnosis and monitoring. Modern technology allows for several types of quantitative imaging metrics for further prognosis and risk stratification as described below.

#### 4.2.1 Total kidney volume (TKV) and height-adjusted TKV (HtTKV)

TKV and HtTKV have been explored extensively as predictive indicators for disease progression in ADPKD. Initially, the Consortium for Radiologic Imaging Studies of Polycystic Kidney Disease (CRISP) hypothesized that TKV effectively reflects the progression of ADPKD (Grantham et al., 2006). Their findings demonstrated that kidney enlargement, driven by cyst expansion, is both continuous and measurable, correlating significantly with declining renal function. Furthermore, accelerated kidney enlargement rates were found to predict more rapid renal functional deterioration (Grantham et al., 2006).

Subsequently, Irazabal et al. introduced a predictive model known as the Mayo Clinic Imaging Classification (MCIC), which utilizes a single measurement of height-adjusted TKV in combination with patient age to estimate the rate of glomerular filtration rate (GFR) decline (Irazabal et al., 2015). The MCIC system classifies ADPKD into two primary classes: class 1 (typical) and class 2 (atypical). Class 1 further stratifies patients aged 15 years or older into five subclasses (1A–1E), based on projected kidney growth rates, where subclasses 1C through 1E denote rapid disease progression (Irazabal et al., 2015). An extensive explanation of the MCIC classification system is detailed elsewhere (Odedra et al., 2023). Analyses of data from the Mayo Clinic Translational Polycystic Kidney Disease Center and CRISP cohorts revealed significant differences in kidney survival across these subclasses. Specifically, patients categorized within subclass 1E exhibited the fastest disease progression and the greatest risk for kidney failure (KF), with hazard ratios indicating incrementally elevated risks from subclasses 1A to 1E (Irazabal et al., 2015). Taken together, MCIC has been approved by the Food and Drug Administration as a specific biomarker for ADPKD and serves as a valuable prognostic indicator of disease severity.

#### 4.2.2 Image texture

Kline et al. examined the structural configurations and varying intensity patterns characteristic of ADPKD using imaging techniques (Kline et al., 2017). Through analysis of MRI-derived texture parameters such as energy, correlation, and entropy, the authors demonstrated associations between these features and percentage changes in eGFR (Kline et al., 2017). These texture metrics were particularly predictive of a ≥30% decline in eGFR and effectively distinguished individuals progressing to CKD stages 3A or 3B. Entropy emerged as the most robust indicator for predicting CKD advancement and significant reductions (≥30%) in eGFR (Kline et al., 2017). Integrating these advanced imaging-derived features with additional non-imaging biomarkers presents a promising strategy for refining personalized clinical interventions in ADPKD management. Nevertheless, the clinical applicability of these findings requires validation through larger prospective cohort studies.

#### 4.2.3 AI in cyst segmentation and advanced imaging biomarkers

Accumulating evidence suggests that AI-assisted cyst segmentation and assessment surpass traditional methods in precision. A previous randomized controlled trial indicated a strong correlation between TKV determined by automated segmentation and manual tracing, exhibiting minimal bias and enhanced accuracy. Such automated approaches effectively mitigate inter-reader variability and minimize human-related errors and biases (van Gastel et al., 2019). Recently, Kline et al. introduced a novel deep-learning (DL) model capable of instance-level cyst segmentation from MRI scans (Gregory et al., 2023). They identified advanced imaging biomarkers including total cyst volume (TCV), height-adjusted renal parenchyma volume (ht-RPV), total cyst number (TCN), and cyst-parenchyma surface area (CPSA). Notably, TCN and CPSA exhibited superior predictive value for disease progression over 8-year and 20-year intervals compared to the conventional height-adjusted TKV metric, highlighting potential enhancements for current prognostic frameworks (Gregory et al., 2023). Raj and colleagues developed a DL-based segmentation model demonstrating robust reliability closely matching ground truth measurements (Raj et al., 2024). Their model accurately segmented kidney volumes, achieving a high correlation coefficient (Pearson’s r = 0.98) between predicted and actual height-adjusted TKV. Furthermore, their prognostic algorithm notably improved predictions of eGFR, achieving a Pearson’s r of 0.86 compared to the MIC) method’s 0.64. Bland–Altman analysis further illustrated that the MCIC tool underestimated absolute eGFR values, whereas the developed DL model displayed narrower variability. Importantly, the authors also trained their algorithm to individually predict CKD stages after 8 years, yielding a weighted f1-score accuracy of 0.851 and an area under the curve (AUC) of 0.972 (Raj et al., 2024). These findings underscore the superiority of DL-based prognostic models over traditional MIC-based methods.

Sheng et al. formulated a versatile DL algorithm compatible with various computed tomography (CT) imaging modalities (Sheng et al., 2025). Their approach effectively processes both Non-enhanced Computed Tomography (NCCT) and Contrast-enhanced Computed Tomography (CCT) scans, ensuring consistent performance and optimized sample usage across different imaging modalities. Their robust localization, segmentation, and TKV estimation model employed a dataset of 200 CT scans, evenly split between NCCT and CCT modalities, sourced from 97 patients diagnosed with ADPKD. The results demonstrated superior performance compared to existing DL architectures, achieving a mean average precision of 95% for automated localization, mean Intersection over Union of 92% for segmentation, and mean R^2^ score of 97% for TKV estimation. Nevertheless, the principal limitation common to these DL-model studies is the relatively small dataset size. Integrative analyses that combine phenotypic and genotypic data are promising avenues for improved comprehension and prognosis of ADPKD. To this end, Sim et al. recently employed a data-driven approach to analyze clinical factors predictive of rapid kidney function decline (RD) using a cohort of 1,744 ADPKD patients (Sim et al., 2024). The developed predictive model identified six key phenotypic clinical parameters significantly associated with rapid renal function decline: age, sex, hypertension, cerebrovascular disease, hemoglobin levels, and proteinuria (Sim et al., 2024).

## 5 Towards precision medicine in ADPKD

### 5.1 Genomic landscape and patient stratification

Recent advances in molecular testing, sequencing technologies, and diagnostic imaging have not only improved the accuracy of ADPKD diagnosis but also paved the way for precision medicine. By integrating genetic, clinical, and imaging data, clinicians can now move beyond disease identification toward individualized risk prediction and tailored therapeutic strategies.

The genotypic diversity of ADPKD lies at the core of precision medicine approach. The difference between the phenotypic manifestation of *PKD1* and *PKD2* translates into a wide variability in clinical outcomes – for example, the median age at ESKD is ∼54 years in *PKD1* patients vs. ∼74 years in *PKD2* patients (Ho et al., 2015). ADPKD follows a “two-hit” mechanism: an inherited germline mutation plus a somatic second hit in renal tubular cells triggers cyst formation. Because somatic mutations can occur stochastically in some nephrons and not others, genetic mosaicism is often observed, contributing to intrafamilial variability (Lanktree and Chapman, 2017). In addition, *modifier genes* can influence disease severity. For instance, patients of African ancestry with high-risk variants in the *APOL1* gene tend to experience faster progression to ESKD, illustrating a polygenic modifier effect superimposed on the primary PKD mutation (Parsa et al., 2013). Such insights have given rise to prognostic algorithms like the PROPKD score, which integrates genotype (e.g., *PKD1* truncating vs. non-truncating vs. *PKD2*) and clinical factors (early hypertension, urologic complications, sex) to predict renal survival (Gall et al., 2016). Patients with high PROPKD scores (e.g., a truncating *PKD1* mutation and early manifestations) have a >90% likelihood of ESKD by age 60, whereas low-risk patients (e.g., *PKD2* carriers) have <20% risk (Gall et al., 2016). This stratification enables personalized management, identifying those who would benefit most from early intervention. Indeed, precision medicine in ADPKD–tailoring therapy to an individual’s genetic risk and disease trajectory – is now entering clinical practice (Lanktree and Chapman, 2017). For example, high-risk patients (such as young *PKD1* truncating mutation carriers with rapidly enlarging kidneys) are prioritized for disease-modifying therapy (e.g., tolvaptan), while low-risk patients may be spared unnecessary exposure (Lanktree and Chapman, 2017).

### 5.2 Mechanistic pathways to targeted therapies

Elucidating ADPKD’s molecular pathology via genomics and epigenomics has opened avenues for targeted interventions beyond conventional supportive care. As discussed in Chapter 2.2, the loss of polycystin function in renal epithelia activates a cascade of cyst-promoting pathways – notably, elevated cyclic AMP signaling, cell-cycle dysregulation, and aberrant metabolism – which can be pharmacologically targeted. The vasopressin V2-receptor antagonist tolvaptan was the first FDA-approved therapy to slow ADPKD progression, and its development was guided by the recognition that cAMP drives cystic proliferation in PKD (Xue et al., 2025). Notably, genotype-driven risk stratification is used to select appropriate candidates for tolvaptan (typically younger *PKD1* patients with rapidly enlarging kidneys), exemplifying genomics-guided therapy in practice. Beyond tolvaptan, a number of pathway-specific drugs (Table 3) are under investigation in clinical trials, often informed by genomic or transcriptomic data. For example, metabolic reprogramming in PKD (partly driven by miR-17 and c-Myc) suggests that agents like metformin (an AMPK activator) could mitigate cyst growth; early-phase trials of metformin in ADPKD have shown it to be safe and hinted at slowed cyst proliferation (Lanktree et al., 2021). Another trial repurposed mTOR inhibitors (rapamycin/everolimus) after genomic studies showed mTOR activation in cystic kidneys, although clinical results were mixed (significant reduction in kidney volume but no clear improvement in GFR) – highlighting the need for better patient selection (e.g., by molecular markers) in such targeted trials.

**TABLE 3:** Summary of gene- and epigenome-based therapeutic approaches in ADPKD.

Therapy/Approach	Target gene/pathway	Mechanism of action	Therapeutic outcome (model/evidence)	Development stage
Tolvaptan	Vasopressin V2 receptor → cAMP pathway	Reduces intracellular cAMP, suppresses cystic fluid secretion and cell proliferation	↓ Kidney growth rate and slower eGFR decline in PKD1 patients	FDA-approved; genotype-guided use
Metformin	AMPK activation → mTOR/energy metabolism	Restores metabolic balance, counteracts miR-17/c-Myc–driven glycolytic shift	Preclinical + early clinical trials show safety and reduced cyst proliferation	Early-phase clinical
mTOR inhibitors (Rapamycin, everolimus)	mTORC1 pathway	Blocks cell-cycle progression and proliferation in cyst epithelium	↓ Kidney volume but limited GFR benefit in trials	Clinical (phase II–III, mixed results)
CRISPR/Cas9 editing	*PKD1*/*PKD2* or regulatory elements	Corrects or disrupts pathogenic alleles; can delete inhibitory miR-17 binding site in *PKD1* 3′UTR	↑ Polycystin-1 levels, ↓ cyst growth in cell and animal models	Preclinical
AAV-mediated gene delivery	*PKD1* C-terminal tail or functional segments	Restores downstream polycystin signaling in renal epithelia	Improved signaling and reduced cystogenesis in animal models	Preclinical
Antisense oligonucleotides (ASOs)	*c-Myc*, *miR-17 cluster*	Suppress cyst-promoting transcription factors or oncogenic miRNAs	↓ Cystogenesis and cell proliferation in mouse models	Preclinical
SMYD2 inhibitors	Histone methyltransferase SMYD2	Block H3 methylation → reduce proliferation signaling	↓ Cyst growth, preserved renal function in Pkd1-mutant mice	Preclinical
EZH2 inhibitors	Histone methyltransferase EZH2	Repress cystic gene transcription via H3K27 methylation blockade	Delayed cyst development in Pkd1-KO mice	Preclinical
HDAC inhibitors	Histone deacetylases (HDACs, e.g., HDAC6, SIRT1)	Restore acetylation and reactivate cyst-suppressor genes	↓ Cyst growth in models; toxicity limits use	Preclinical
DNA methylation inhibitors (5-azacytidine)	*MUPCDH* promoter hypermethylation	Reactivates MUPCDH expression → slows cyst cell proliferation	Restored expression *in vitro*; potential urine methylation biomarker	Experimental
miR-192/194 mimics	miR-192/194 family (loss in ADPKD)	Reinstate anti-fibrotic and anti-EMT functions	↓ Cyst growth in Pkd1-KO mouse model	Preclinical
anti-miR-17 oligonucleotide	miR-17 (from miR-17–92 cluster)	Blocks miR-17 → restores PPAR-α and mitochondrial function	↓ Cyst growth and inflammation in mouse and human cyst cultures	Preclinical
miR-214 agonist/mimic	miR-214 (upregulated protectively)	Enhances negative feedback on TLR4 inflammatory axis	Reduces macrophage-driven inflammation and cyst growth in mice	Preclinical
Nanocarrier-based epigenetic delivery	EZH2/SMYD2 inhibitors in kidney-targeted vehicles	Concentrates epigenetic drug in renal tissue to limit toxicity	Enhanced local effect in preclinical models	Conceptual/preclinical

In parallel, gene-based therapies are on the horizon, aiming to correct or mitigate the root genetic defect in ADPKD. The large size of *PKD1* (coding ∼4300 amino acids) and the necessity to target kidney cells specifically make gene therapy challenging, but progress is being made. Novel CRISPR/Cas9 genome editing approaches have been applied in PKD models, for example, to knock out pathogenic alleles or disrupt deleterious regulatory elements. One creative strategy used CRISPR to delete a microRNA binding site in the *PKD1* 3′UTR, thereby preventing miR-17 from downregulating the wild-type *PKD1* transcript (Xue et al., 2025). This *in situ* genome editing approach led to increased polycystin-1 levels and ameliorated cyst growth in cell models (Xue et al., 2025). Other approaches include AAV-mediated gene delivery of the polycystin-1 C-terminal tail (to rescue downstream signaling defects), and antisense oligonucleotides to modulate disease genes. For example, an antisense oligo against c-Myc (a transcription factor driving cyst proliferation) has shown benefit in an orthologous ADPKD mouse, reducing cystogenesis (Xue et al., 2025). Similarly, antisense therapy against miR-17 (as noted earlier) is being pursued (Xue et al., 2025). While these advanced therapies are still in preclinical or early-phase development, they exemplify the move toward *precision medicine* – intervening at the individual molecular defect level.

### 5.3 Epigenomic pathways to targeted therapies

Epigenomic discoveries have yielded a new class of therapeutic targets: epigenetic enzymes that can be “drugged” to alter cyst cell behavior (Table 3). One promising target is *SMYD2*, mentioned in Chapter 3 as an enhancer of proliferative signalling enhancer in cyst-lining cells. In a *Pkd1*-mutant mouse model, genetic KO of *SMYD2* resulted in reduced cyst growth and preserved renal function (Xu et al., 2022). Pharmacologic SMYD2 inhibitors also slowed cyst expansion in preclinical studies (Xu et al., 2022). Similarly, inhibition of EZH2 in *Pkd1*-KO mice delayed cyst development (Xu et al., 2022). These findings position histone methyltransferases as attractive therapeutic targets. However, given that such enzymes are ubiquitous, selective delivery is crucial to avoid systemic toxicity. Innovative strategies are being explored, such as kidney-targeted nanocarriers loaded with epigenetic drugs (e.g., EZH2 or SMYD2 inhibitors), to concentrate the effect in renal cystic tissue (Giblin et al., 2025). Likewise, the reversible nature of epigenetic marks raises the prospect of using DNA methylation inhibitors or HDAC inhibitors to re-activate cyst-suppressor genes. For instance, preclinical studies have tested pan-HDAC inhibitors and found some efficacy in reducing cyst growth, although side effects remain a concern (Xue et al., 2013). As of now, no epigenetic therapies have reached clinical trial for ADPKD, but these approaches are under active development (Giblin et al., 2025).

Building on the epigenetic mechanisms previously discussed, emerging research illuminates specific therapeutic targets and biomarker applications. While DNA methylation patterns are now recognized as critical in ADPKD pathogenesis, translating these insights into clinical applications remains a key priority. One striking example is the *MUPCDH*: silencing of the *MUPCDH* promoter by hypermethylation is associated with accelerated cyst growth (Xu et al., 2022). Patients whose urine-derived DNA shows fully methylated *MUPCDH* have a faster increase in total kidney volume, suggesting that urinary *MUPCDH* methylation status could serve as a prognostic biomarker for disease progression (Xu et al., 2022). This is clinically significant, as a noninvasive urine DNA methylation assay might help identify rapid progressors. Experimentally, demethylating treatment (5-azacytidine) can restore *MUPCDH* expression and slow the proliferation of cystic cells in culture, pointing to a potential therapeutic angle (Xu et al., 2022; Mi Woo et al., 2015).

### 5.4 Targeted therapies miRs in ADPKD

Genome-wide miRNA profiling of ADPKD tissues identified the miR-192/miR-194 family as epigenetically suppressed in cystic epithelia (Xu et al., 2022). These miRNAs are normally expressed in tubular cells and help maintain epithelial characteristics by targeting pro-fibrotic and pro-EMT factors (e.g., ZEB2 and N-cadherin) (Xu et al., 2022). In ADPKD, *miR-192/194* loci become hypermethylated, and their expression declines, especially in advanced cyst-laden kidneys (Kim et al., 2019). The loss of miR-192/194 lifts repression on EMT drivers, potentially facilitating cyst expansion and interstitial fibrosis. Encouragingly, reconstitution of miR-192/194 has shown therapeutic effects: injection of miR-192/194 mimics in a *Pkd1*-KO mouse model significantly reduced kidney cyst growth (Kim et al., 2019). This suggests that restoring beneficial microRNAs (or blocking their epigenetic silencing) could slow the disease – a novel strategy quite different from conventional therapies (Kim et al., 2019). On the other hand, some microRNAs act as *pathogenic drivers* and thus become targets for inhibition. A prime example is miR-17 (from the miR-1792 cluster), which is markedly upregulated in ADPKD cyst cells (Hajarnis et al., 2017). MiR-17 promotes aberrant cell proliferation partly by repressing PPAR-α and shifting metabolism away from mitochondrial oxidative phosphorylation (Hajarnis et al., 2017). In mouse models, genetic deletion of miR-17∼92 or treatment with an anti-miR-17 oligonucleotide dramatically attenuated cyst development and preserved renal function (Hajarnis et al., 2017). Notably, human ADPKD cyst cultures treated with anti-miR-17 also showed reduced proliferation (Hajarnis et al., 2017). These findings position the miR-17 family as a promising therapeutic target (Hajarnis et al., 2017). Conversely, certain miRNAs appear to function in *negative feedback* to mitigate disease. For instance, miR-214, which is derived from the long non-coding RNA *DNM3OS*, is upregulated in the renal *interstitial cells* surrounding cysts (Lakhia et al., 2020). MiR-214 is induced by inflammatory signals (TLR4/IFN-γ/STAT1) in the cyst microenvironment and in turn directly suppresses *Tlr4*, thus dampening macrophage-driven inflammation (Lakhia et al., 2020). Deletion of miR-214 in a mouse model led to heightened pericystic inflammation and accelerated cyst growth, whereas the presence of miR-214 restrains pro-cystic inflammation (Lakhia et al., 2020). This suggests miR-214 is a protective factor, and therapies that *boost* certain anti-inflammatory miRNAs might be beneficial (Lakhia et al., 2020). Together, such discoveries underscore that ADPKD’s epigenome is not merely a bystander but an active regulator of disease course – and a rich source of biomarkers. For example, differential methylation patterns in cell-free DNA from blood are being explored as minimally invasive biomarkers to predict cyst burden and therapeutic response (Xu et al., 2022). Likewise, urinary exosome profiling has revealed that ADPKD patients excrete lower levels of polycystin-1/2 proteins compared to healthy individuals, and exosomal PC1 or PC2 levels inversely correlate with kidney volume (Ho et al., 2015). The urine exosomal PC1:TMEM2 ratio in particular has been proposed as a diagnostic and prognostic indicator (Ho et al., 2015). As these examples show, integrating epigenomic and proteomic markers can augment traditional imaging in monitoring ADPKD.

### 5.5 Outlook: from omics to personalized care

The convergence of genomic and epigenomic research in ADPKD is accelerating the realization of personalized medicine for this disease. Genetic testing is increasingly used in clinical practice to confirm ADPKD in atypical cases and to inform prognostication (Lanktree and Chapman, 2017). With the broader availability of panel sequencing, even patients without *PKD1/PKD2* mutations (so-called “DNA-negative” ADPKD) can sometimes be reclassified (e.g., mutations in *GANAB* or *DNAJB11* leading to PKD phenocopies), which guides appropriate management (Cornec-Le Gall et al., 2018). Meanwhile, emerging biomarkers derived from epigenomic alterations (such as urine methylation signatures or circulating microRNAs) may soon supplement imaging and kidney volume in tracking disease progression and drug response. These biomarkers could enable early feedback on whether a therapy is hitting its target pathway in a given patient–a key aspect of precision treatment. Ultimately, the goal is a therapeutic arsenal tailored to each ADPKD patient’s molecular profile. In the near future, an ADPKD patient might receive a combination of treatments: for example, a V2-receptor blocker to reduce cAMP (tolvaptan), plus a miR-17 inhibitor to re-engage metabolic pathways, and perhaps an epigenetic modulator to reactivate a cyst-suppressing gene – each chosen based on that patient’s genotype/epigenotype and risk markers. Ongoing clinical trials and translational studies are integrating these insights, bringing us closer to truly personalized therapy for ADPKD. The hope is that by targeting the right mechanism in the right patient at the right time, we can significantly improve outcomes in ADPKD, slowing cystic disease before irreversible damage accumulates and tailoring interventions to maximize efficacy while minimizing unnecessary treatment burden.

### 5.6 Future perspectives

Looking toward the future, several key research priorities emerge. First, the integration of multi-omics approaches, combining genomics, epigenomics, transcriptomics, and other omics, will provide a more comprehensive understanding of disease heterogeneity and identify novel therapeutic targets. Second, the development of kidney-targeted drug delivery systems will be crucial for translating epigenetic therapies into clinical practice while minimizing systemic toxicity. Third, the expansion of genetic testing accessibility and the incorporation of rare causative genes into routine clinical panels will improve diagnostic yield and enable more precise classification of ADPKD variants. The ultimate goal is the realization of truly personalized medicine for ADPKD patients, where therapeutic decisions are guided by an individual’s complete molecular profile, encompassing genetic variants, epigenetic modifications, biomarker profiles, and imaging characteristics. This approach promises to optimize treatment efficacy while minimizing unnecessary exposure to interventions, fundamentally improving outcomes for the millions of individuals affected by this progressive disease. As we advance toward this precision medicine future, continued collaboration between scientists, clinicians, and technology developers will be essential to translate these promising discoveries into tangible benefits for ADPKD patients. The foundation established by current research provides a robust platform for the next generation of therapeutic innovations that may finally alter the natural history of this devastating disease.

## 6 Conclusion

ADPKD represents a clear example of how advances in genomic and epigenomic research are transforming our understanding of inherited disease mechanisms and therapeutic approaches. This comprehensive review has highlighted the complex molecular landscape underlying ADPKD pathogenesis, stressing how genetic mutations in *PKD1* and *PKD2*, along with rare causative genes, interact with sophisticated epigenetic regulatory networks to drive cystogenesis and disease progression. The integration of cutting-edge diagnostic technologies, from LRS that overcomes traditional limitations in *PKD1* analysis to AI-assisted imaging biomarkers that provide unprecedented precision in disease monitoring, is revolutionizing clinical practice. Emerging epigenetic insights, including the roles of DNA methylation patterns, histone modifications, and non-coding RNAs in modulating disease severity and progression rates complement these technological advances. The identification of biomarkers such as urinary methylation signatures and circulating microRNAs offers the promise of non-invasive monitoring tools that could transform patient management. The development of risk stratification algorithms like the PROPKD score, which integrates genetic and clinical factors to predict disease trajectory, exemplifies how molecular insights can guide personalized treatment decisions. High-risk patients with *PKD1* truncating mutations and rapid disease progression can now be identified early and prioritized for disease-modifying therapies, while low-risk patients may be spared unnecessary interventions. The therapeutic landscape is also expanding beyond conventional supportive care to include pathway-specific interventions targeting the molecular drivers of cystogenesis. From vasopressin V2-receptor antagonists guided by cAMP pathway insights to emerging epigenetic therapies targeting histone methyltransferases and DNA methylation machinery, these approaches represent a fundamental shift toward mechanism-based treatment strategies. Gene therapy and genome editing technologies, while still in early development, hold promise for addressing the root genetic defects underlying the disease.
